# A 12-Week Randomized Double-Blind Placebo-Controlled Clinical Trial, Evaluating the Effect of Supplementation with a Spinach Extract on Skeletal Muscle Fitness in Adults Older Than 50 Years of Age

**DOI:** 10.3390/nu13124373

**Published:** 2021-12-06

**Authors:** Silvia Pérez-Piñero, Vicente Ávila-Gandía, Jacobo A. Rubio Arias, Juan Carlos Muñoz-Carrillo, Pilar Losada-Zafrilla, Francisco Javier López-Román

**Affiliations:** 1 Sports Physiology Department, Faculty of Health Sciences, Campus de los Jerónimos s/n, UCAM Universidad Católica San Antonio de Murcia, E-30107 Murcia, Spain; sperez2@ucam.edu (S.P.-P.); jcmunoz@ucam.edu (J.C.M.-C.); plosadazafrilla@gmail.com (P.L.-Z.); jlroman@ucam.edu (F.J.L.-R.); 2 Department of Education, University of Almería, E-04120 Almería, Spain; jararias@ual.es; 3 Primary Care Research Group, Biomedical Research Institute of Murcia (IMIB-Arrixaca), E-30120 Murcia, Spain

**Keywords:** natural spinach extract, healthy aging, muscle health, randomized trial, strength training

## Abstract

The aim of a 12-week randomized double-blind placebo-controlled study was to assess the effect of daily supplementation with a natural extract of *Spinacia oleracea* L. (4 × 500 mg capsules/day; total 2 g per day) combined with a moderate-intensity training program (1 h session/3 times a week) on skeletal muscle fitness in adults over 50 years of age. Muscle strength assessed by isokinetic and isometric dynamometry improved significantly in the experimental (*n* = 23) and the placebo (*n* = 22) groups, but the magnitude of improvement was higher in the experimental group, with between-group differences in almost all variables, including isokinetic at 60° s^−1^ in knee extension, peak torque (*p* < 0.007); total work per repetition maximum (*p* < 0.009); isokinetic at 180°s^−1^ in knee extension, peak torque (*p* < 0.002); total work (*p* < 0.007); total work per repetition maximum (*p* < 0.005); average power (*p* < 0.027); isometric in knee extension, peak torque (*p* < 0.005); and average peak torque (*p* < 0.002). Similar findings were observed for muscle quality. Changes in quality of life (SF-36) were not found, except for improvements in the role physical (*p* < 0.023) and role emotional (*p* < 0.001) domains, likely as a result of the physical training sessions. A nutritional survey did not revealed changes in dietary habits. No adverse events were recorded. In subjects over 50 years of age, moderate-intensity strength training combined with daily supplementation for 12 weeks with a natural extract of *Spinacia oleracea* L. improved muscle-related variables and muscle quality. Maintaining muscle health is a key component of healthy aging.

## 1. Introduction

Healthy aging is a progressively important concept that refers to promoting and maintaining the functional capacity that allows well-being in old age [1]. Maintaining skeletal muscle fitness is a key component to reduce loss of muscle mass and to prevent adverse health outcomes associated with decreased physical function and reduced mobility [2,3]. Remarkably, exercise and physical activity are countermeasures against muscle aging and have been shown to attenuate age-related decreases in skeletal muscle performance [4]. There is evidence that an active lifestyle supported by appropriate dietary nutrition is crucial to preserve strong, healthy, skeletal muscles [5].

Physical exercise, dietary interventions, and nutritional supplementation have been advocated as main interventions for treating or preventing loss of skeletal muscle mass and strength. Resistance training is a form of exercise that improves muscle strength and endurance and is progressively gaining attention and interest as a method used to improve physical fitness and functional performance in elderly people [6]. The main objective of resistance training programs should be to gradually overload in order to achieve positive adaptations in both the improvement of muscle mass and muscle functioning, taking into account the physiological processes of aging [7]. Recommendations of resistance training programs for older adults include 2–3 sets of 3–4 polyarticular exercises, involving large muscle groups at intensities of 70–85%, of 1 repetition maximum (1RM), 2 to 3 times per week [8]. In addition, when strength training is scheduled in periods, by executing each repetition with high velocity, greater improvements in functional performance are achieved [9,10]. According to recommendations by the National Strength and Conditioning Association [11], resistance training programs for older adults should follow the principles of individualization, periodization, and progression.

In relation to diet as a modifiable factor, a relationship between healthier diets with nutrient adequacy and better muscle mass outcomes has been suggested [12]. A systematic review based on the data of 23 studies provided strong evidence for a link between healthier diets and lower risk of decline in physical performance, supporting whole-diet interventions for the prevention of progressive loss of skeletal muscle mass [13]. Moreover, it has been shown that a diet is a determinant that contributes to maintain quality of life in older age. In a systematic review of 15 studies, healthy dietary patterns and adherence to healthy diets, such as the Mediterranean diet, showed an association with improvements in different domains of self-reported quality of life in older adults [14]. In an analysis of data of 2203 participants, of the French ”Supplémentation en Vitamines et Minéraux Antioxydants” cohort, aged 45–60 years, a one-point increase in a five-component healthy lifestyle index (weight, smoking status, physical activity, alcohol consumption and diet) was related to a 11% higher probability of healthy aging, defined as absence of chronic diseases and function-limiting pain, good physical and cognitive functioning, functional independence, no depressive symptoms, and good social and self-perceived health [15].

Numerous studies related to the consumption of a large variety of nutrients/nutraceuticals have shown beneficial effects on different aspects of skeletal muscle health and exercise performance (strength and endurance capacity) through antioxidant, anti-inflammatory, or anabolic mechanisms, some of which are still poorly understood [16,17]. In this context, dietary macro- and micronutrients, vitamins, minerals, long-chain polyunsaturated fatty acids, phytochemicals, triterpenoids, and probiotics are among the list of compounds for which a preventive effect on muscle atrophy via the attenuation of muscle protein degradation and the promotion of protein synthesis and myogenesis have been suggested [18,19].

Spinach extract is another widely used food supplement or ergogenic aid, characterized by a high content of phytoecdysteroids, particularly 20-hydroxyecdysone (20E), which have been shown to increase strength and muscle mass during resistance training, to reduce fatigue and to facilitate recovery [20]. Doses higher than 5 µg/kg body weight were reported as active while lower doses did not result in anabolic activities [21]. Although several proteolytic systems are involved in protein turnover in skeletal muscle, recent studies suggested that the ubiquitin-proteasome system (UPS) represents the major intracellular proteolysis machinery responsible for the degradation of major contractile proteins, and contributes significantly to muscle atrophy and sarcopenia in both animal and human models [22,23]. Ecdysteroids also have a protein-synthesis effect and influence on lipid and carbohydrate metabolism [18]. One of the most interesting properties of ecdysteroids in mammals is their purported adaptogenic and non-androgenic anabolic properties [24]. Although several studies failed to identify a frankly anabolic effect on total muscle mass by ecdysteroids [25,26,27], the signaling pathway of the ubiquitin-proteasome was suggested to be involved in the ecdysteroids-induced increase in protein synthesis [28,29,30], together with a downstream activation of the PI3K/Akt pathway [31]. Thus, the compounds that are found in a high percentage in spinach may have beneficial actions in maintaining skeletal muscle fitness in older adults.

The ergogenic potential of dietary nitrate (NO_3_^−^) supplementation has shown beneficial effects on muscle contractility and muscle efficiency, as well as to improve muscle endurance during weightlifting exercise in healthy adults [32]. Moreover, dietary NO_3_^−^ can improve exercise tolerance in older patient populations, which are affected by reduced O_2_ delivery to skeletal muscles [32]. In a large cohort of 1420 older women of ≥70 years of age, the relationship between total nitrate intake and muscle function measures was analyzed [33]. Each standard deviation higher nitrate intake (31.2 mg/kg) was associated with stronger grip strength and faster timed-up-and-go, so that increasing dietary nitrate, especially through vegetable consumption, may be an effective way to limit age-related declines in muscle function [34].

On the other hand, muscle strength training combined with food supplementation strategies seems to increase the benefits, and become a doubly effective intervention to promote healthy aging in adults over 50 years of age [35,36,37]. However, the existing evidence is still limited, and further studies on the association of dietary interventions and exercise training are needed, in which clinical recommendations for the prevention or treatment of decline in muscle mass and quality could be based. Therefore, the present clinical trial was conducted to evaluate the efficacy of spinach extract supplementation on skeletal muscle fitness in healthy adults over 50 years of age performing strength training. It was hypothesized that supplementation with a natural spinach extract of *Spinacia oleracea* L. combined with 12 weeks of a physical exercise program would be associated with improvements in muscle health contributing to healthy aging.

## 2. Materials and Methods

### 2.1. Design and Participants

This was a single-center, randomized, double-blind, placebo-controlled study with two parallel arms conducted at the Health Sciences Department of Universidad Católica San Antonio de Murcia (UCAM), in Murcia, Spain. The study began in 14 September, 2020, and finished in 8 June 2021. The primary objective of this study was to assess the efficacy of daily consumption of a spinach extract for 12 weeks on in adults older than 50 years of age who simultaneously participated in a 12-week physical exercise resistance training program on skeletal muscle fitness. Secondary objectives included changes in body composition and quality of life, as well as a nutritional evaluation and assessment of safety of the study product. Participants were mainly recruited by advertising the study through mass media and talks in women’s centers, elderly community centers, and neighbourhood associations. Eligible subjects were Caucasian men and postmenopausal women aged between 50 and 75 years, with a body mass index (BMI) less than 32 kg/m^2^, and who did not perform scheduled physical exercise on a weekly basis. Exclusion criteria were subjects with the presence of chronic diseases that would prevent the execution of a physical exercise training program (e.g., moderate/severe chronic obstructive pulmonary disease, currently treated ischemic heart disease, arrhythmia, diabetes, arthritis, etc.); absolute or relative contraindications for physical exercise training, according to criteria established by the American College of Sports Medicine (ACSM); alcohol consumption (>20 g/day); consumption of a functional food or supplement that may affect body composition within the previous 6 months; pregnant or breast-feeding women; hypersensitivity or intolerance to any component of the study product; inability to understand the informed consent, and ineligibility as judged by the investigators.

The study protocol was approved by the Ethics Committee of Universidad Católica San Antonio de Murcia (code CE032003, approval date 27 March 2020) (Murcia, Spain) and was registered in the ClinicalTrials.gov (accessed on 1 November 2021) (NCT04612127). Written informed consent was obtained from all participants.

### 2.2. Intervention and Study Procedures

Participants were randomly assigned using a simple randomization procedure to the experimental group (supplementation with the spinach extract nutritional product) or the control group (supplementation with placebo) with the Epidat 4.2 software program. A physical resistance training program was combined with dietary supplementation in all participants regardless of the assigned group. Randomization was performed by an independent researcher.

The active product was an extract of *Spinacia oleracea* L. (edible leaf) (Spisar™, EUROMED, S.A., Mollet del Vallès, Barcelona, Spain), prepared using an eco-friendly and proprietary water-based extraction process (Pure-Hydro Process™). High-performance liquid chromatography (HPLC) analysis of the batch used for the study reported 1600 ppm of 20-hydroxyecdysone. The composition per 100 g included carbohydrates 29.3 g, protein 20.4 g, fat < 0.5 g, dietary fiber 9.6 g, sodium 10.3 g NaCl, vitamin C < 15 mg, calcium 39.5 mg, iron 34.3 mg, potassium 11.4 mg, and total sugars 20.7 mg. The content of nitrates was 7088 mg NO_3_/kg and total amino derivatives expressed as pipecolic acid (died extract) 2.9%.

Subjects in the intervention group were instructed to take four capsules a day (4 × 500 mg each), in two divided doses, at breakfast and lunch for 12 weeks (total amount of nitrate consumption 14.18 mg/day). Subjects in the control group received identical-appearing placebo capsules (maltodextrin) and followed the same regimen. All participants were instructed to refrain from making changes in their dietary habits, particularly flavonoid-containing foods (coffee, tea, chocolate, nuts, etc.) and from starting or modifying habitual hormone and medical treatments. The importance of maintaining dietary habits during the study was emphasized. Enrollment in physical exercise training activities other than the resistance training program included in the study was not allowed.

Participants were visited at baseline (visit 1) and at 90 days (visit 2, final visit). During the study period, they were also requested to be adherent with the exercise training sessions, which were scheduled 3 times a week, and the loss of three training days was only allowed. At baseline, the written informed consent was obtained, fulfilment of the inclusion criteria was checked, and the study product was provided. Body composition, muscle function, muscle quality, quality of life, and nutritional assessment were evaluated at visits 1 and 2. Tolerability and safety of the product was evaluated at visit 2.

### 2.3. Physical Resistance Training Program

The physical resistance training program was based on individualized and progressive polyarticular strength exercises involving the main muscle groups with moderate intensity (60–75% 1RM) aimed to increase muscle mass. Participants performed the 1RM from the load-velocity relationship based on the movement velocity test for half-squat on a Smith machine (Technogym, Cesena, Italy). The first load was set at 5 kg corresponding to the barbell without weights, followed by the addition of different loads (10 kg) in each repetition until the mean propulsive velocity (MPV) was <0.5 m/s as reported in other studies [38,39], with at least 3 min resting (or 5 min for heavier loads) between repetitions. Then, load steps of 1 to 5 kg until the 1RM was determined and subsequently determined the 1RM prediction equations from the load-velocity profile of each participant.

Training was performed 3 days a week, on alternate days, 1 h per session, and in the same time slots. Exercises performed at each session were barbell squat with guide bar (Bodytone Multipower Evolution Series EB01), and quadriceps extension, “jalon” pulldowns, and chest press using weightlifting machines (Marcy Eclipse HG3000 Compact Home Gym station). Sessions started with a 5-min warm-up period on a stationary bike (45 w, 70–75 rpm), followed by the four scheduled exercises (3 sets of 10 repetitions were performed with 5 reserve repetitions of each exercise, with a minute and a half of active rest between sets), and finished with stretches and scapular mobility exercises for 10 min. All training sessions were supervised by a qualified sport-training monitor. Maximal dynamic force was measured after 2 weeks of familiarization with the exercises in order to determine the training loads for each participant. Loads were adjusted every week by the control of the mean propulsive velocity (MPV) using a linear velocity transducer (Chronojump Boscosystem, Barcelona, Spain), the cable of which was attached to the extreme of the bar and showed displacement time data at a frequency of 1000 Hz. During the training sessions, execution of the barbell squat was performed with a 1-s stop between the eccentric–concentric phases to eliminate the energy accumulated during sitting down, ensuring MPV reliability. Subjects were also told that the concentric phase should be performed as rapid as possible. The intensity and control of training was measured by the rating of perceived exertion (RPE), a modified 10-point Borg scale (0: very light stress—rest; 10: very hard stress) [40]. Progressive training intensity was established on an individual basis and as participants were told that they should keep 5 reserve repetitions for each set, subjective perceived exertion always remained around 5–6 at maximum in the 10-point Borg scale. This control of exercise intensity allowed the subjects to start each training session without a state of fatigue, thereby achieving greater adherence to training.

### 2.4. Study Variables

Clinical variables included age, gender, height (Seca 700, GmbH, Hamburg, Germany), weight (TANITA BC-420MA, Tanita Corporation, Tokyo, Japan), and systolic and diastolic blood pressure (BP). Blood pressure was using an OMRON M6 AC blood pressure monitor (Omron Healthcare España). Body composition was measured by total body dual-energy X-ray absorptiometry (DEXA) to measure fat mass, lean mass, muscle mass, and appendicular skeletal muscle mass (ASM) of the dominant leg using the Norland XR-46 bone densitometry system.

Muscle function of knee extension torques was measured by isokinetic dynamometry of the dominant leg using isokinetic dynamometer Biodex System 3 (Biodex Medical Systems, Shirley, NY, USA) at 60° and 180° s^−1^ based on 5 repetitions. After warming up for 5 min on an ergometric bicycle, subjects were seated with a 90° hip flexion on the knee module, aligning the knee movement axis with the dynamometer axis. The resistance pad was placed on the distal two third of the tibia. Range of motion was set between 0 and 90° of knee flexion. Variables measured were peak torque isokinetic value (expressed as Newton meters (Nm)), total work (TW) measured in Joules, average power measured in watts, and total work per repetition maximum measured in Joules. In the isometric dynamometry (at 90°, the lever did not allow movement of the leg and the force trying to perform a complete extension of the knee was measured. The force was maintained for 5 s with a resting period of 30 s between repetitions. Three repetitions after a one warm-up test were performed. Variables measured were peak isometric torque (Nm) and average peak isometric torque (Nm). Handgrip strength was measured in the sitting position using Takei 5401 Grip-D digital dynamometer (Takei Scientific Instruments, Tokyo, Japan) with the maximum grip (expressed in kg). Three repetitions were performed with the right hand and with the left hand, and the mean of the two best for each hand was used. Muscle quality was calculated taking into account the muscle mass between the peak torques. In this study, it was estimated in four different ways, taking into account the muscle mass obtained by DEXA and ASM by the isokinetic value of peak torque at 60° s^−1^ extension of the dominant leg and the isometric value of maximum torque, and expressed as N × m/kg.

The health-related quality of life (HRQoL) was measured using a validated Spanish version of the short form-36 health survey (SF-36) [41]. The questionnaire covers eight dimensions, including physical functioning, the role physical, bodily pain, general health, vitality or energy/fatigue, social functioning, role emotional, and mental health. For each dimension, items are recodified, averaged together, and transformed into a 0–100 scale, with higher scores representing better health.

The 24-h recall method was used for the dietary survey. Data were recorded over a 3-day period at baseline and at the end of the intervention (two weekdays and one weekend day). Dietary intake was analyzed using DietSource^®®^ v3.0 software, and the variables registered were energy intake (Kcal) and absolute and relative calories of macronutrients (carbohydrates, lipids and proteins). DietSource^®®^ is a versatile software program used for different purposes, including nutrient intake calculation, dietary assessment, preparation of diets based on food composition tables, consultation of nutrition recommendations and dietary guidelines, etc.

The returned capsules at visit 2 (end of study) were checked and counted for adherence with the study product. Adherence was defined as consumption of at least 80% of capsules, so that only 72 capsules could be left corresponding to 18 days out of 90 days of consumption. Participants were directly asked for adverse events. Overnight fasting venous blood samples were drawn from a peripheral vein at baseline and at the end of the study for normality of safety variables. Laboratory data were complete hemogram, liver function including bilirubin, aspartate aminotransferase (AST), alanine aminotransferase (ALT), gamma-glutamyl transpeptidase (GGT) and lactate dehydrogenase (LDH), and renal function tests including serum creatinine levels and blood urea nitrogen.

### 2.5. Statistical Analysis

The sample size was calculated according to the peak torque at 60° s^−1^ in isokinetic dynamometry as the main variable of the study. Considering a standard deviation of peak torque of 15 Nm [39] for a precision of 11 Nm with an alpha risk of 5% and statistical power of 80%, 23 subjects in each group was needed, increasing to 25 subjects per group assuming a 10% loss to follow-up.

The analysis was based on the per-protocol (PP) data set corresponding to those participants who met the inclusion criteria and finished the study at 12 weeks. Categorical variables are expressed as frequencies and percentages, and quantitative variables as mean ± standard deviation (SD). The chi-square (χ^2^) test or the Fisher’s exact test was used for the comparison of categorical variables between the study groups, and the Student’s *t* test for the comparison of continuous variables when comparing groups at baseline. Changes of variables in the study groups over the course of the study were analyzed with the analysis of variance (ANOVA) for repeated measures with two study factors: within subject factor (baseline and 12 weeks) and between-subject factor (intervention: active product and placebo) for paired data. Post-hoc analyses were performed with the Bonferroni correction. An additional comparison of muscle function and muscle quality between men and women was performed. Statistical significance was set at *p* < 0.05. The SPSS version 25.0 (IBM Corp., Armonk, NY, USA) was used for data analysis.

## 3. Results

### 3.1. General Characteristics of the Study Population

**Figure 1:**
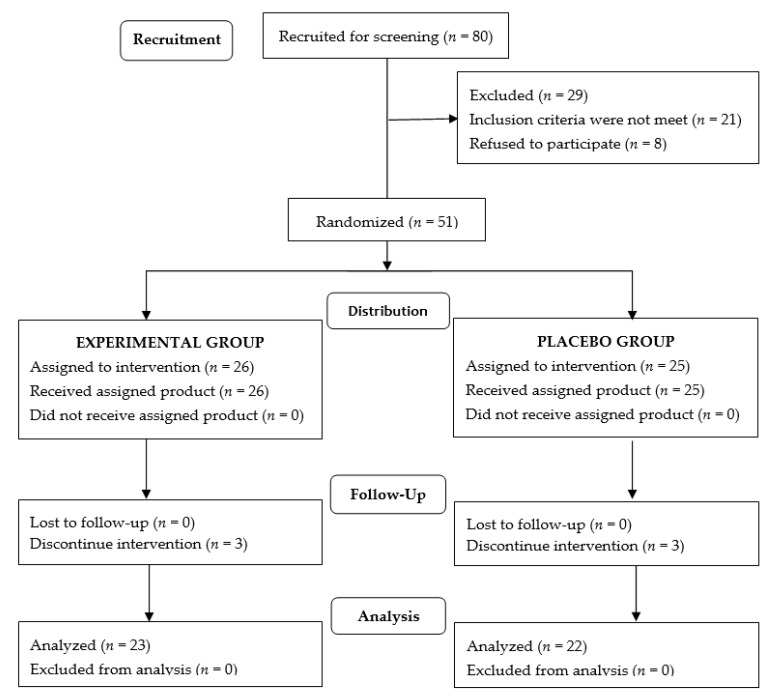
Flow chart of the study population.

Of a total of 80 voluntary subjects, 29 were excluded because the inclusion criteria were not met (*n* = 21) or refusal to participate (*n* = 8). The remaining 51 subjects were randomized (26 to the experimental group and 25 to the placebo group), but 3 subjects in each group were lost to follow-up mostly due to impossibility of attending physical training sessions (e.g., life-change, moving to another city). Therefore, the final study population included 45 subjects (23 in the experimental group, 22 in the placebo group), 8 men, and 37 women, with a mean age of 58.9 ± 6.1 years. Training protocols were the same for the participants assigned to each study group. The flow chart of the study population is shown in Figure 1. There were no statistically significant differences between the study groups in the baseline characteristics of participants (Table 1). Moreover, there were no significant differences in the main baseline variables between subjects who completed the study and those who were lost to follow-up. An analysis limited to male participants did not show statistically significant differences in any baseline variables between the experimental and the placebo groups (data not shown).

### 3.2. Body Composition

Baseline data of the two study groups were similar. The consumption of the spinach extract or placebo for 12 weeks was associated with a reduction of fat mass and an increase in lean and muscle mass. In both study groups, differences in these parameters at the end of the study as compared with baseline were statistically significant when body composition was evaluated by DEXA. However, between-group differences were not statistically significant (Table 2).

### 3.3. Muscle Function

Baseline data of the two study groups were similar. Muscle function improved significantly at the end of the study as compared with baseline both in the experimental and the placebo groups, but the magnitude of improvements was higher in the experimental group (Table 3).

**Figure 2:**
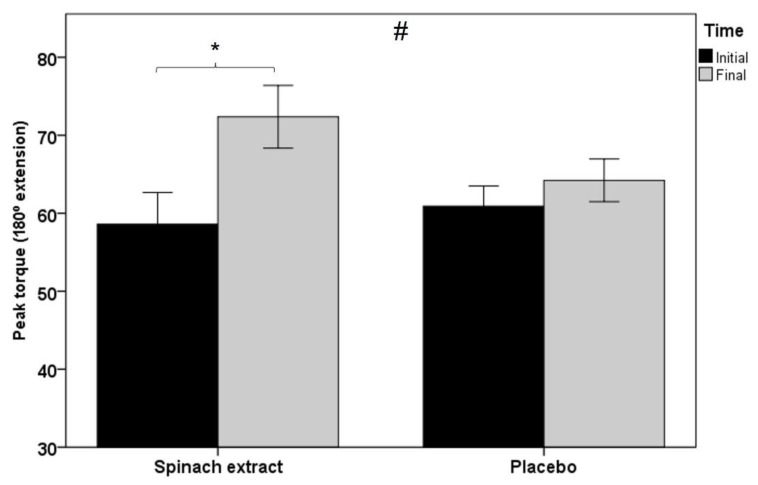
Within-group and between-group differences in peak torque at 180° s^−1^ knee extension in isokinetic dynamometry testing (error bars ± 1 SD; * *p* = 0.001; ^#^ *p* = 0.002).

**Figure 3:**
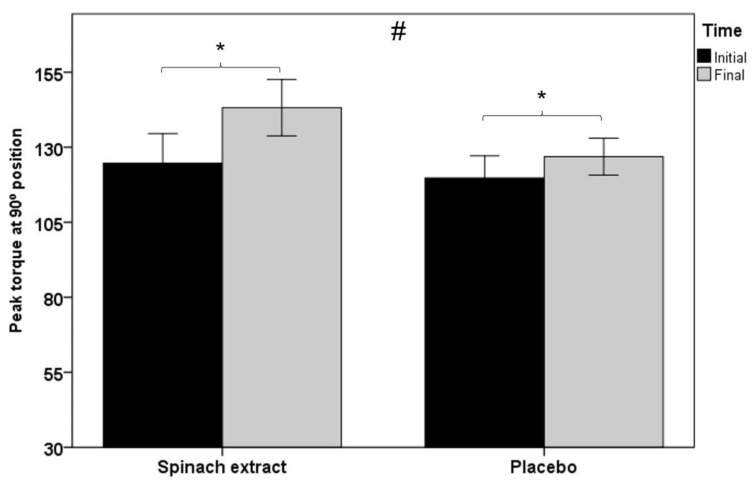
Within-group and between-group differences in peak torque at 90° position in isometric dynamometry testing (error bars ± 1 SD; * *p* = 0.001 experimental group and *p* = 0.012 control group; ^#^ *p* = 0.005).

As shown in Table 3, statistically significant between-group differences were observed for all muscle function variables, except for total work and average power in isokinetic dynamometry testing at 60° s^−1^ knee extension, although at 180° s^−1^ knee extension, between-group comparisons were statistically significant for all variables. Figure 2 shows within-group and between-group differences in favor of the experimental group in peak torque at 180° s^−1^ knee extension in isokinetic dynamometry (*p* < 0.001), and Figure 3 significant differences in both groups of peak torque in isometric dynamometry (*p* < 0.001 in the experimental group and *p* < 0.012 in the placebo group).

Differences in handgrip strength were not significant. Maximal dynamic force (1RM) increased in both groups, but between-group differences did not reach statistical significance.

### 3.4. Muscle Quality

Baseline data of the two study groups were similar. Muscle quality showed a significant improvement in both study groups at the end of the study as compared with baseline as estimated by the relationship between muscle mass obtained by DEXA and ASM and peak torque value of isokinetic at 60° s^−1^ extension of the dominant leg and isometric dynamometry studies. As shown in Table 4, within-group and between-group differences were statistically significant in all variables.

### 3.5. Changes in Muscle Parameters According to Gender

An additional analysis of muscle mass of the lower limbs, isokinetic and isometric strength, and muscle quality by gender was performed (Table 5).

In men, consumption of the active product induced a higher increase in muscle mass in the lower limb than placebo, resulting in higher muscle strength and a greater increase in muscle quality.

In women, improvements in muscle mass were similar in both study groups, although gains in muscle strength were superior in the experimental group than in women treated with placebo. Gains in muscle quality were higher in the experimental group but statistically significant differences by gender were not found.

### 3.6. Health-Related Quality of Life

The use of the study products, either active spinach supplementation or placebo was associated with general improvements in the perception of HRQoL, with significant within-group differences at the end of the study as compared with baseline in the two domains of the role physical and role emotional (Table 6).

### 3.7. Nutritional Survey

Assessment of the nutritional survey showed that subjects in both study groups maintained their dietary habits throughout the study period, and no differences were obtained in any of the macronutrients groups and total calories intake (Table 7).

The study products were well tolerated and no adverse effects were recorded. All participants consumed a percentage higher than 80% of capsules. Results of laboratory tests were within the normal ranges.

Finally, the percentage of patients with sarcopenia at the end of the study decreased from 65.2% to 52.2% in the experimental group and from 77.3% to 59.1% in the placebo group.

## 4. Discussion

Physical activity and nutrition are two modifiable lifestyle factors with direct impact on the health of older people, with interventions targeting muscle fitness and dietary nutrition aimed to preserve healthy aging. This study was conducted under the perspective of improving muscle health in people over 50 years of age with the combination of a muscle strength physical training program and the consumption of a dietary supplement of a natural spinach extract. In relation to the primary outcome, the use of the spinach supplement improved muscle strength evaluated by isokinetic and isometric dynamometry. Subjects assigned to the active product showed improvements of muscle function of greater magnitudes than those treated with placebo, with between-group significant differences in all variables in dynamometry testing at 180° s^−1^ and isometric dynamometry at 90° position. Therefore, consumption of a spinach extract for 12 weeks was associated with improvement in muscle strength measured by dynamometry of the dominant leg. Handgrip strength, however, did not appear to be improved by the study product. An interesting finding of the study was the improvement in muscle quality in the group assigned to the active product to a greater extent than subjects treated with placebo, so the consumption of the spinach extract produced a higher increase in muscle quality than that associated with the physical training in the absence of active supplementation. In relation to muscle quality, we have not found current evidence regarding whether an improvement of 0.1 in muscle mass/peak torque is biologically important. In our opinion, gains in muscle quality in the intervention group is very positive. Better muscle quality is an important contributor of muscle health and healthy aging [4,5].

Moreover, there was a significant decrease of fat mass in both study groups and an increase in muscle mass. As the modifications appeared in both groups, it is apparent that they are the result of the physical training program carried out during the study. We found a significant decrease in fat mass in both groups, but this change was not clinically significant (2.1% in the placebo group and 1.4% in the experimental group) [43]. A possible explanation for the absence of a clinically significant reduction is that the diet was not controlled, and on the other hand, the use of an exercise protocol aimed at fat loss [44], as has been seen in other studies, in which a reduction in fat mass has been achieved with the combination of strength and endurance training and an individualized hypocaloric diet [44]. However, the objective of the product was not the reduction of body fat, so that the decrease in body fat is probably due to the effect of physical exercise.

A complementary and exploratory analysis of muscle mass-related variables by gender was performed, although it should be noted that the analysis of gender effects for men should be interpreted cautiously because there were only eight men in the study. In men, consumption of the active product caused a higher increase in muscle mass as compared with placebo. The strength developed by the experimental group was higher than the strength that could be developed by the placebo group; an increase in muscle quality was also observed. Analysis of these variables in women revealed that increases in muscle mass of the lower limb were similar in the two study groups, but improvements in muscle strength were higher as compared with placebo [45]. This finding may suggest that in women, gains in muscle strength may be due to improvement of the neuromuscular components, as opposed to a greater gain in muscle mass in men [46]. Moreover, muscle quality gains in the experimental group were similar in men and women. This may be due to the fact that muscle quality is the ratio between the strength developed in the lower body and muscle mass, components developed in different proportions by women and men, respectively [47].

After training, there was an increase of muscle mass of 1.5% in the experimental group and 1.1% in the placebo group, but statistically significant between-group differences were not observed. However, when muscle mass of the dominant lower extremity was assessed, there was only a significant increase in the experimental group. The lack of greater increases in muscle mass may be explained by the type of training based on four exercises, two of the legs, one of the back, and one of the chest, so that greater effect on the lower limbs would had been expected. Previous studies, however, have reported that increase in muscle thickness in the upper body are greater and occur earlier compared to the lower extremity, during the first 12 weeks of a total body dynamic resistance training program [46,48]. Because of the characteristics of our physical exercises, it may be possible that the effects on muscle mass, and secondarily of the active supplement, could have been optimized by the use of training protocols with greater volume loads and repetitions [49].

It should be noted that 82.6% of participants in the experimental group and 81.8% in the placebo group were women. Both men and women respond to training with an improvement of strength and muscle mass [46,50]. However, the response to training in men and young women (25 years of age) is higher given that older adults (approximately 69 years old) showed a slow hypertrophy of type 1 muscle fibers after a period of strength training [51], and in this respect, the size and contractile material of type 2 fibers decrease with age [52], so that with the type of training used in the study and the characteristics of the study population (people older than 50 years), there could have been an attenuation of the effects of the supplementation product on muscle mass.

Moreover, although similar improvements are observed in elderly men and women, the relative improvement in upper-body strength is greater in women [45], whereas greater hypertrophy and low-body strength are shown in males [47]. These observations are in line with the study by Tracy et al. [51], who assessed the effects of strength training of short duration (9 weeks) in 65- to 75-year-old men and women, and found similar improvements in muscle mass and muscle quality, although older men appeared to have a greater capacity for absolute strength and muscle mass gains than older women. Improvements observed in muscle quality may have been primarily acquired by muscle mass increases in men and neuromuscular factors in women [46].

In relation to the changes observed in HRQoL, improvements were observed in all domains of the SF-36 questionnaire, with statistically significant differences within groups at the end of the study compared to baseline in the role physical with physical exercise being the most plausible cause. Although in both groups there are significant differences in the evolution of the role emotional, there is a greater increase in the role emotional, in favor of the spinach extract treatment, would be in line with the adaptive properties of ecdysteroids as observed in the study by Franco et al. [24]. In addition, no changes in macronutrient intake were observed during the study period. No adverse events associated with daily ingestion of the active product were observed for 12 weeks, and laboratory test results were within normal limits.

The present results indicate that in adults older than 50 years of age, performing physical exercise associated with the consumption of spinach extract supplement was associated with beneficial effects on muscle health. Accordingly, it may be postulated that the use of the food supplement may exert an overall “all-body strengthening” adaptogenic activity and may be beneficial for improving some manifestations of age-related skeletal muscle mass atrophy through the maintenance of muscle health and muscle, which are crucial for healthy aging. Therefore, the spinach extract supplementation would be particularly useful for improving muscle fitness, in the context of prevention of adverse muscle changes associated with muscle atrophy in middle-aged subjects, in association with physical exercise. In contrast to the results of an early study by Wilborn et al. [27], in which no ergogenic effects were observed, our study did identify relevant ergogenic properties. A more recent study of Isenmann et al. [20] found significant increases in muscle mass in those participants given two to eight capsules of ecdysterone (6 mg per capsule) combined with the amino acid leucine (100 mg per capsule) daily. Our data do not show such a marked anabolic effect and this discrepancy can potentially be explained by a lower daily dose, a different experimental protocol, and/or some interference with leucine. The potential synergism between hydroxyecdysone and leucine may have played a role in the more obvious changes in body composition reported by Isenmann et al. [20]. Leucine induces the activation of translation factors such as PI3K-Akt (similarly to ecdysone) and promotes translation initiation and protein synthesis. Daily doses of 0.6 g leucine can induce robust muscle protein synthesis, as observed in the study by Wilkinson et al. [53]. On the other hand, the nitrate content of the natural spinach-based product could also have been contributed to the positive effects on muscle health. Systematic reviews and meta-analyses have shown that dietary nitrate is a useful nutritional strategy to optimize muscular performance during resistance and endurance exercises in different training modalities [54,55,56].

The present findings, however, should be interpreted, taking into account the limitations of the study, including the exploratory nature of the trial, the reduced sample size, and the treatment period of 12 weeks only. All female participants were postmenopausal women, but the use of hormone replacement treatment was not specifically recorded as an exclusion criterion. However, all study participants were interviewed regarding what medications and pharmacological treatments and/or natural products were taken, and this information was supervised by the doctor (specialist in sports medicine) who made final decisions regarding the eligibility of participants. Moreover, potential gender differences in muscle-related variables is an aspect that may be evaluated in further studies, because of the small number of men included in the present exploratory analysis.

Although the nutritional survey of macronutrients at the end of the study showed that subjects in both study groups had maintained their dietary habits, dietary intake during the protocol was not controlled. It can be argued that it is difficult to isolate an effect of spinach extract on muscle strength because of the absence of vegetable, fruit, or vitamin intake evaluations, but the presence of a control group, the double-blind design of the study, and the dietary control based on strong recommendations to maintain dietary habits and results of the nutritional survey, minimizes this limitation. It is recommended to research spinach extracts in further clinical trials with a larger study population, with higher training intensities and/or prolonged training periods, with subjects stratified by gender, to further clarify the role of supplementation with *Spinacia oleracea* L. in the promotion of healthy aging.

## 5. Conclusions

In the pursuit of healthy aging, especially as adults begin to age, this study focused on the combination of moderate-intensity strength training and dietary supplementation with a natural extract of *Spinacia oleracea* L. The main findings showed significant improvements in muscle strength assessed by isokinetic and isometric dynamometry and muscle quality, as well as a reduction of fat mass and an increase in muscle mass. Improvements in the role physical and role emotional domains of HRQoL may be attributed to benefits of the physical training program. Preserving muscle health, in particular, skeletal muscle fitness in old age, is imperative for the maintenance of functional independency and quality of life.

## Figures and Tables

**Table 1:** Baseline characteristics of participants.

Variables	Experimental Group (*n* = 23)	Placebo Group (*n* = 22)	*p* Value
Gender			
Men	4	4	0.894
Women	19	18	
Age, years, mean ± SD	59.2 ± 5.6	58.6 ± 6.6	0.750
Weight, kg, mean ± SD	69.3 ± 8.2	68.0 ± 9.7	0.633
Body mass index, kg/m^2^, mean ± SD	25.9 ± 3.0	26.1 ± 3.5	0.819
Percentage of fat mass, mean ± SD	40.3 ± 5.0	41.8 ± 5.3	0.341
SBP, mmHg, mean ± SD	128.0 ± 20.4	123.1 ± 14.3	0.353
DBP, mmHg, mean ± SD	82.6 ± 11.9	80.3 ± 9.1	0.473
Men (SMI ≤ 7.0 kg/m^2^), * mean ± SD, (*n*)	5.66 ± 0.00 (1)	6.46 ± 0.31 (2)	0.283
Women (SMI ≤ 6.0 kg/m^2^), * mean ± SD, (*n*)	5.44 ± 0.44 (14)	5.40 ± 0.55 (15)	0.793

SD: standard deviation; SBP: systolic blood pressure; DBP: diastolic blood pressure; SMI: skeletal muscle index. Data expressed as frequencies and percentages unless otherwise stated. * Cut-off points of the European Working Group on Sarcopenia in Older People 2 (EWGSOP2) [42].

**Table 2:** Changes in body weight and body composition measured by DEXA at the end of the study after 12 weeks of food supplementation with a spinach extract or placebo.

Variables	Experimental Group (*n* = 23)			Placebo Group (*n* = 22)			Time × Product Interaction *p* Value
	Baseline	End of Study	Within-Group *p* Value	Baseline	End of Study	Within-Group *p* Value	
Body weight, kg	69.3 ± 8.2	69.0 ± 8.4	0.374	68.0 ± 9.7	67.7 ± 9.7	0.361	0.975
DEXA analysis							
Fat mass, kg	27.8 ± 4.1	27.4 ± 4.3	0.050	28.3 ± 4.9	27.7 ± 4.8	0.014	0.615
Lean mass, kg	39.0 ± 6.6	39.6 ± 6.8	0.001	37.7 ± 7.4	38.1 ± 7.3	0.020	0.254
Muscle mass, kg	38.8 ± 6.9	39.4 ± 6.9	0.001	37.5 ± 7.5	37.9 ± 7.4	0.016	0.429
ASM, dominant leg, kg	6.2 ± 1.3	6.3 ± 1.3	0.013	6.0 ± 1.4	6.0 ± 1.3	0.143	0.463

DEXA: dual-energy X-ray absorptiometry; ASM: appendicular skeletal muscle mass.

**Table 3:** Changes of muscle function of knee isokinetic and isometric dynamometry and handgrip strength at the end of the study after 12 weeks of food supplementation with a spinach extract or placebo.

Variables	Experimental Group (*n* = 23)			Placebo Group (*n* = 22)			Time × Product Interaction *p* Value
	Baseline	End of Study	Within-Group *p* Value	Baseline	End of Study	Within-Group *p* Value	
Isokinetic dynamometry							
At 60° s^−1^ knee extension							
Peak torque, Nm	91.6 ± 28.1	107.2 ± 26.6	0.001	93.6 ± 16.9	101.4 ± 18.0	0.001	0.007
Total work for 1RM, J	83.3 ± 23.3	101.5 ± 24.7	0.001	85.2 ± 13.6	94.3 ± 20.0	0.001	0.009
Total work, J	390.3 ± 115.3	474.4 ± 118.3	0.001	384.7 ± 85.6	445.6 ± 92.4	0.001	0.289
Average power, W	53.6 ± 17.8	63.4 ± 17.2	0.001	54.2 ± 9.8	60.3 ± 13.2	0.001	0.109
At 180° s^−1^ knee extension							
Peak torque, Nm	58.6 ± 19.5	72.4 ± 19.3	0.001	60.9 ± 12.2	64.2 ± 12.9	0.143	0.002
Total work for 1RM, J	62.2 ± 22.3	79.2 ± 23.5	0.001	62.5 ± 12.3	68.4 ± 14.3	0.034	0.005
Total work, J	285.3 ± 100.9	363.3 ± 104.5	0.001	287.7 ± 61.0	317.3 ± 66.8	0.020	0.007
Average power, W	93.3 ± 35.6	115.1 ± 31.9	0.001	94.4 ± 22.3	102.2 ± 24.6	0.080	0.027
Isometric dynamometry							
At 90° knee position							
Peak torque, Nm	124.7 ± 47.3	143.2 ± 45.1	0.001	119.7 ± 34.7	126.9 ± 28.9	0.012	0.005
Average peak torque, Nm	118.7 ± 44.5	136.4 ± 44.7	0.001	114.4 ± 34.2	121.0 ± 29.4	0.008	0.002
Handgrip strength, kg							
Right hand	25.7 ± 8.2	26.4 ± 7.1	0.193	24.8 ± 8.7	24.9 ± 7.9	0.823	0.449
Left hand	23.3 ± 7.4	25.4 ± 7.4	0.004	23.3 ± 7.8	24.3 ± 6.9	0.203	0.247
Maximal dynamic force (1RM), kg	43.2 ± 13.8	60.1 ± 16.7	0.001	41.4 ± 14.0	57.3 ± 15.1	0.001	0.729

**Table 4:** Changes of muscle quality at the end of the study after 12 weeks of food supplementation with a spinach extract or placebo.

Variables	Experimental Group (*n* = 23)			Placebo Group (*n* = 22)			Time × Product Interaction *p* Value
	Baseline	End of Study	Within-Group *p* Value	Baseline	End of Study	Within-Group *p* Value	
DEXA study							
Muscle mass/peak isokinetic torque (60° s^−1^ extension), N × m/kg	2.4 ± 0.7	2.7 ± 0.5	0.001	2.5 ± 0.4	2.7 ± 0.4	0.002	0.024
Muscle mass/peak isometric torque, N × m/kg	3.2 ± 1.0	3.6 ± 0.9	0.001	3.2 ± 0.7	3.3 ± 0.6	0.034	0.021
ASM dominant leg							
Muscle mass/peak isokinetic torque (60° s^−1^ extension), N × m/kg	15.0 ± 4.2	17.2 ± 3.6	0.001	16.0 ± 2.6	17.1 ± 2.7	0.003	0.022
Muscle mass/peak isometric torque, N × m/kg	20.2 ± 6.1	22.9 ± 5.7	0.001	20.2 ± 4.5	21.2 ± 3.8	0.046	0.018

DEXA: dual-energy X-ray absorptiometry; ASM: appendicular skeletal muscle mass.

**Table 5:** Gender differences in gains of muscle mass, strength, and muscle quality at the end of the study after 12 weeks of food supplementation with a spinach extract or placebo.

Variable	Gender	Study Group	Baseline	Final End of Study	∆	Time *p* Value	Time × Product *p* Value	Time × Product × Gender *p* Value
Muscle mass of the dominant lower limb, kg	Men	Experimental	8.0 ± 1.7	8.3 ± 1.7	0.3	0.030	0.041	0.015
		Placebo	7.7 ± 2.3	7.7 ± 2.1	−0.05	0.455		
	Women	Experimental	5.8 ± 0.8	5.9 ± 0.8	0.1	0.005	0.712	
		Placebo	5.6 ± 0.6	5.7 ± 0.7	0.1	0.020		
Peak isokinetic torque (60° s^−1^ extension), Nm	Men	Experimental	96.9 ± 36.7	114.4 ± 35.8	17.5	0.001	0.067	0.925
		Placebo	112.7 ± 25.7	121.9 ± 21.8	9.2	0.05		
	Women	Experimental	90.5 ± 27.1	105.7 ± 25.3	15.2	0.001	0.024	
		Placebo	89.4 ± 11.4	96.9 ± 14.0	7.5	0.001		
Peak isometric torque (90° position), Nm	Men	Experimental	127.0 ± 61	152.0 ± 61.0	25	0.001	0.023	0.085
		Placebo	161.0 ± 55.0	160 ± 43.0	−1	0.895		
	Women	Experimental	124.0 ± 46.0	141.0 ± 43.0	17	0.001	0.05	
		Placebo	111.0 ± 21.0	119.0 ± 20.0	8	0.005		
Muscle quality DEXA (muscle mass/peak isometric torque), N × m/kg	Men	Experimental	2.52 ± 1.14	3.0 ± 1.12	0.48	0.01	0.036	0.333
		Placebo	3.20 ± 0.90	3.20 ± 0.82	0	0.993		
	Women	Experimental	3.36 ± 0.93	3.77 ± 0.83	0.41	0.001	0.106	
		Placebo	3.21 ± 0.66	3.41 ± 0.55	0.20	0.022		

**Table 6:** Changes in HRQoL (SF-36 questionnaire) at the study after 12 weeks of food supplementation with a spinach extract or placebo.

SF-36 domain	Experimental Group (*n* = 23)			Placebo Group (*n* = 22)			Between-Group *p* Value
	Baseline	End of Study	Within-Group *p* Value	Baseline	End of Study	Within-Group *p* Value	
Physical functioning	88.9 ± 9.6	90.9 ± 5.8	0.277	85.2 ± 13.0	88.2 ± 10.1	0.111	0.696
Role physical	89.1 ± 7.9	91.7 ± 6.3	0.023	85.2 ± 10.5	88.9 ± 7.1	0.002	0.518
Bodily pain	79.9 ± 19.2	81.1 ± 17.6	0.636	72.1 ± 23.3	73.2 ± 21.9	0.813	0.871
General health	66.1 ± 11.2	69.0 ± 14.5	0.210	73.6 ± 13.8	74.1 ± 17.6	0.832	0.465
Vitality or energy/fatigue	60.9 ± 16.0	62.8 ± 13.5	0.422	65.2 ± 13.4	65.0 ± 4.1	0.927	0.530
Social functioning	87.0 ± 16.2	91.3 ± 16.2	0.184	88.6 ± 17.6	92.0 ± 17.9	0.306	0.839
Role emotional	70.8 ± 11.1	78.0 ± 8.2	0.001	79.6 ± 10.5	83.9 ± 8.6	0.003	0.118
Mental health	66.6 ± 11.5	71.8 ± 11.6	0.049	73.1 ± 11.2	74.0 ± 17.7	0.732	0.249
Self-reported health transition	43.5 ± 21.6	41.3 ± 16.2	0.573	52.3 ± 7.4	37.5 ± 16.8	0.001	0.026

**Table 7:** Results of nutritional survey.

Variables	Spinach Extract Group (*n* = 23)		Placebo Group (*n* = 22)	
	Baseline	End of Study	Baseline	End of Study
Calories (Kcal)	1960.9 ± 39.9	2054.5 ± 45.3	2030.9 ± 45.0	1856.6 ± 49.6
Carbohydrates, g	256.9 ± 27.5	267.9 ± 25.3	213.1 ± 30.9	228.8 ± 44.5
Fat, g	66.5 ± 4.9	71.2 ± 9.4	91.3 ± 17.5	64.3 ± 15.5
Proteins, g	83.7 ± 4.2	85.6 ± 7.3	89.2 ± 3.9	91.5 ± 5.2

## Data Availability

Study data are available from the principal investigator (F.J. López-Román) upon request.
